# Feasibility of Video Clip Analysis on Effect of Botulinum Toxin-A Injection for Post-Stroke Upper Limb Spasticity

**DOI:** 10.3390/toxins5050983

**Published:** 2013-05-10

**Authors:** Woo-Jin Kim, Witsanu Kumthornthip, Byung Mo Oh, Eun Joo Yang, Nam-Jong Paik

**Affiliations:** 1 Department of Rehabilitation Medicine, Seoul National University Bundang Hospital, Seongnam 463-707, South Korea; E-Mails: cyctaz@hanmail.net (W.-J.K.); graceloves@gmail.com (E.J.Y.); 2 Department of Physical Medicine and Rehabilitation, Haeundae Paik Hospital, Inje University of Medicine, Busan 612-896, South Korea; 3 Department of Rehabilitation Medicine, Siriraj Hospital, Mahidol University, Bankok 73170, Thailand; E-Mail: wkumthornthip@yahoo.com; 4 Department of Rehabilitation Medicine, Seoul National University Hospital, Seoul 110-744, South Korea; E-Mail: keepwiz@gmail.com; 5 Department of Rehabilitation Medicine, Seoul National University College of Medicine, Seoul 110-799, South Korea

**Keywords:** botulinum toxin A, injection, spasticity, upper limb, video, stroke

## Abstract

Existing functional evaluation tools do not accurately reveal the improved function following botulinum toxin A (BTX-A) injection for post-stroke upper limb spasticity. With the aim of developing an alternate method of measuring functional improvement following BTX-A injection, this study tested the feasibility, validity and reliability of video clip analysis performed by the clinicians. Seventy-nine patients administered BTX-A due to post-stroke upper limb spasticity, were retrospectively evaluated using video clip analysis. Pre- and post-injection video clips recorded at 1-month intervals were randomly allocated and sent to three blinded physician evaluators who were asked to choose the one that seemed more improved in terms of hand motion and associated upper limb reaction during gait. The three physicians chose the post-injection video clip as depicting improved hand motion (82.3%, 79.7%, and 72.2%) and associated upper limb reaction during gait (73.4%, 70.9%, and 70.9%). Kappa and intraclass correlation coefficient as a measure of interrater reliability among the three physicians was 0.86 and 0.79 for the hand, and 0.92 and 0.92 for associated upper limb reaction during gait, respectively. The percent overall agreement of the physicians was 78.1% and 71.7% for hand function and associated upper limb reaction, respectively. Retrospective pre- and post-BTX-A injection video clip analyses is a clinically feasible alternative method to evaluate the improvement following BTX-A injection for post-stroke upper limb spasticity, especially in busy clinical practice setting.

## 1. Introduction

Stroke is a leading cause of mortality and long-term morbidity [1]. Stroke affects 700,000 Americans a year [2] and many stroke survivors experience residual disability with limited functional recovery of the upper limbs. Apart from loss of function due to paresis, spasticity may be one of major contributing factors to disability [3]. A majority of stroke survivors exhibit persisting motor deficits and various manifestations of the upper motor neuron syndrome caused by spasticity [1]. 

Spasticity is defined as a velocity dependent increase in muscle resistance to imposed movement [4] and the hyper-excitability of stretch reflexes [5]. Spasticity develops in various upper motor neuron disorders such as stroke, spinal cord injury, multiple sclerosis and cerebral palsy [6]. The incidence of post-stroke spasticity is estimated to be about 19% to 38% [2]. Spastic hypertonia causes restricted joint range of motion, excessive force on joints, and pain, leading to compensatory abnormal posture, functional limitations in activities of daily living and ambulation, and increased care giver burden [7]. 

Treatment goals for spasticity include pain relief, reducing muscle frequency of spasm and involuntary movement associated reactions, decreasing care giver burden, improving body image by enhancing aesthetic and postural appearance, preventing contractures and deformities, and improving active function. Active functions that are desirable to improve are dexterity, reaching, activities of daily living such as washing, dressing, eating, drinking, sexual activity, and mobility functions such as transfer and gait.

Botulinum toxin type A (BTX-A), one of the most potent and yet safe biological toxins, blocks acetylcholine release from motor neuron and lead to reversible paralysis of the injected spastic muscle with its effect lasting for two to four months [5]. It has been widely used in clinics to focally reduce the spastic limbs and is effective in improving the joint range of motion motor function [5], and is also cost-effective for treating post-stroke limb spasticity compared to oral anti-spastic drugs [8]. 

There are many approaches measuring spasticity. These include indirect measures, predominantly using scales such as the Modified Ashworth Scale, which has documented validity and reliability [9], and direct measures in which physical properties are mechanically or neurophysiologically quantified [10]. Global functional assessment scores such as modified Barthel Index (MBI) and functional independence measure (FIM) are also used to measure functional improvement following BTX-A injection. However, these measuring tools were mostly unsuccessful in demonstrating significant functional improvements following BTX-A injection [3]. Such findings imply that reduction in spasticity does not always accompany increased function, and the evaluation tools used to date to measure functional status do not reflect the improved active or passive function after BTX-A injection. To overcome this limitation, a more flexible outcome measure following BTX-A injection was introduced, the Goal Attainment Scale (GAS), which adapts not only passive and active functions, but also across the domains of impairment, disability, and participation [11]. Discrepancy in goal setting between the physician and the patient and the time-consuming process of goal setting and scale rating makes GAS less feasible for application in routine clinical practice [12,13]. Also, it measures goal achievement rather than functional improvement. 

We investigated the use of video clips as a new evaluation tool for analyzing pre- and post-BTX-A injection. Our aim was to determine whether this video clip analysis could be used as an alternative method for evaluating the effects of BTX-A injection for post-stroke upper limbs spasticity by demonstrating its validity and reliability.

## 2. Patients and Methods

### 2.1. Patients

Medical records and video clips of pre- and post-BTX-A injection into spastic upper limbs of 79 stroke patients at the Department of Rehabilitation of (blinded) hospital between May 2005 and August 2009 were retrospectively collected. Data on gender, age, diagnosis, stroke onset, mean duration from stroke onset to BTX-A injection, degree of spasticity, injection site, and dosage were reviewed. This study was approved by the institutional review board of the (blinded) hospital. 

### 2.2. Botox Injection

One vial (100 IU of Botox; Allergan, USA or 500 IU of Dysport; Ipsen, UK) was reconstituted with 4.0 mL or 5.0 mL of normal saline. Total doses administered to the spastic upper limb were 100 to 400 IU for Botox and 500 to 2000 IU for Dysport. Injected forearm and upper arm muscles were biceps brachii (BB), brachioradialis (BR), brachialis, flexor carpi radialis (FCR), flexor carpi ulnaris (FCU), flexor digitorum profundus (FDP), flexor digitorum superficialis (FDS), flexor pollicis longus (FPL), and adductor pollicis (AP). The dosage and muscle selection was individualized based on the severity and distribution of the involved hemiplegic spastic limb. Procedure was carried out using portable electromyography guided and/or nerve stimulation guided by the one same physician. 

### 2.3. Functional Measures and Video Analysis

**Figure 1:**
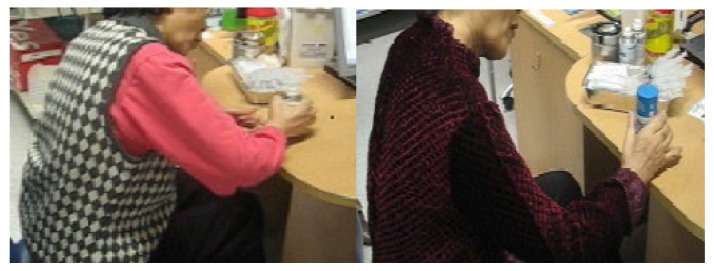
Pre and post-operative captured images of video-clip, demonstrating improvement of the cylindrical grasp and release on the right.

Before injection and 1 month following injection, functional measurement was performed using modified Rankin Scale (mRS) [14], Korean version of modified Barthel Index (K-MBI), Brunnstrom Stage [15], modified Ashworth Scale (MAS) [10] and Fugl-Meyer Score [16]. For video recording, active and passive motion of elbow flexion, extension, pronation and supination, and hand motion during performance of hand grasp and release, object gripping and shifting, as well as posture of upper limb during gait were recorded in routine clinical sessions. The camera was fixed within 1 meter from the affected side of the patient during physical exam which was carried out by single physician, and all patients were positioned at the side of desk in same manner (Figure 1). During this 1-month period, medications including anti-spasticity medication were unchanged and all patients were on their routine rehabilitative treatment or follow up as usual, and no additional therapy was provided. After trimming the pre- and post-injection video clips to avoid exposure of recognizable chronological order of pre- and post-status to ensure that the evaluators were blinded, the clips from each patient were randomly allocated according to the random sample chart prepared in advance by a third party (Figure 1). The evaluators were asked to compare and choose the video clip that they thought represented greater improvement in terms of hand motion and posture of upper limb during gait. Three board certified physicians from different hospitals evaluated the video clips and their results were sent back and analyzed in terms of validity and interrater reliability. For validity analysis, the post-injection video was operationally regarded as a more improved one. 

### 2.4. Statistics

Interrater reliability or agreement between physicians A, B, and C were evaluated with kappa coefficients, interclass correlation coefficient (ICC) and percent overall agreement. The degree of agreement between 79 ratings of the three physicians of upper limb motion and gait associated upper limb posture were separately calculated. The kappa coefficient of agreement is expressed as *k* = (*P*_o_ − *P*_c_)/(100 − *P*_c_), and is standardized to lie on a −1 to 1 scale, where 1 is perfect agreement. Kappa coefficients and ICC values were interpreted based on recommendation by Cicchetti [17] (<0.40, poor; 0.40–0.59, fair; 0.60–0.74, good; and ≥0.75, excellent). 

## 3. Results

The demographic data of the 79 patients (55 males) are summarized in Table 1. The mean age at the time of the injection was 60 ± 17 years. The mean duration from stroke onset to BTX-A injection was 11 ± 9 weeks (ranging from 2 to 44 weeks). The mean mRS of the patients at the time of injection was 4.0 and the mean Brunnstrom stage in the arm and hand were 2.8 and 2.5, respectively. The mean upper limb MAS before BTX-A injection was 2.0. Pre-injection MBI showed average score of 26.0 and FMA was 17.3 for the affected upper limb and 52.6 for the unaffected upper limb. The mean duration from the injection to follow-up ranged from 3 to 12 weeks, with a mean duration of 4.21 weeks. The mean mRS and MAS at the post-injection follow-up were 3.5 and 1.6, respectively. The mean Brunnstrom stage in the arm and hand at the follow up were 3.4 and 3.1, respectively. Post injection MBI showed mean score of 57.1 and FMA was 23.0 on the affected upper limb and 61.6 on the unaffected upper limb. Overall, all of the items including mRS, Brunnstrom stage score in the affected arm and hand, MAS, as well as the functional evaluation scores improved, but only Brunnstrom stage score in the affected hand and MAS showed statistical significances. 

**Table 1:** Patient characteristics.

	Pre	Post	*p* Value
Age (years)	60 ± 17		
M:F	55:24		
Time from stroke onset to BTX-A injection (weeks)	11 ± 9		
mRS	4	3.5	0.335
B stage (arm)	2.83	3.39	0.144
B stage (hand)	2.43	3.08	0.036
MAS	2	1.58	0.011
FMA (affected)	17.3	23	0.161
FMA (unaffected)	52.6	61.6	0.116
MBI	26	57.1	0.335

B stage, Brunnstrom stage; MAS, modified Ashworth Scale; FMA, Fugl-Myer Assessment; MBI, modified Barthel Index; mRS, modified Rankin Scale.

### Validity and Reliability Analysis

Rating of the video clips by physicians A, B, and C was validated for each hand motion and gait associated hand postural appearance over post-injection video clip operationally defined as being the more improved one (Figure 1). Physician A matched 65 and 58 for hand motion and gait associated hand posture, and mismatched 14 and 21 for each of hand motion and gait associated hand posture, with validity of 82.3% for hand motion and 73.4% for gait associated hand posture. Physician B matched 63 and 56 and mismatched 26 and 23, with validity of 79.7% for hand motion and 70.9% for gait associated hand posture. Physician C matched 57 and 56 and mismatched 22 and 23 with validity of 72.2% and 70.9% (Table 2). 

**Table 2:** Validity of the video recordings.

	Physician A	Physician B	Physician C
Hand motion	82.3%	79.7%	72.2%
Gait associated hand posture	73.4%	70.9%	70.9%

Kappa coefficients for hand motion and gait associated hand posture were 0.86 and 0.92, respectively, both falling in the excellent range. Intraclass correlation coefficient (ICCs) were also high, falling within the excellent range for both the hand motion (0.79) and gait associated hand posture (0.92). The percent overall agreement between physicians A, B, and C for hand motion and gait associated hand posture were 78.1% and 71.7%, respectively (Table 3). 

**Table 3:** Interrater agreement between the three evaluators.

	kappa	ICC	Overall agreement (%)
Hand motion	0.86	0.79	78.1
Gait associated hand posture	0.92	0.92	71.7

ICC intraclass correlation coefficient.

## 4. Discussion

The study was not designed to demonstrate the beneficial effect of BTX-A injection for post-stroke spastic upper limb function, but rather to demonstrate the feasibility of physician video clip analysis as an alternative outcome measure for assessing improvement following BTX-A injection by estimating its validity and reliability.

Current management of post-stroke upper limb spasticity can often be challenging and unsatisfactory for patients and clinicians, despite the ease of diagnosis. Goals of spasticity management for post-stroke upper limb spasticity include increase in passive/active range of motion, decrease in frequency of muscle spasm and involuntary movements, pain relief, attaining better hygiene, improving splint wear, and improvement of dexterity and other functional activities such as self care and eating [3]. In addition to standard conservative measures such as positioning, stretching, and exercise, treatment options include physical agents, oral antispastic drugs, and neuromuscular blockade by local injection of phenol or botulinum toxin and intrathecal baclofen [3]. Oral antispastic medications such as baclofen, dantrolene and diazepam are non-selective, and tolerance develops that often requires an increase in dose to sustain a required clinical response [18], which can increase the incidence of unwanted systemic side effects such as weakness, sedation, and dry mouth. Most importantly, these medications are still unsatisfactory for relief of spasticity. 

Spasticity control using focal injections such as phenol or BTX-A injection enhances the effects of conventional rehabilitative treatments and reduces the use of systemic anti-spastic medications. Furthermore, benefits of BTX-A injection for the treatment of post-stroke upper limb spasticity have been established with a statistically significant decrease of spasticity following its use when measured with MAS [19], without the adverse effects. However, the benefits of BTX-A injection for improvement of upper limb active function has not been fully demonstrated, and to date has shown statistically non-significant improvements. Usually these effects are measured using functional scales such as MBI, FIM, or a neurological scale such as the Fugl-Meyer score. However, such measurement scales reflect functional improvement on just one or two items following BTX-A injection, and any improvement in these items may be diluted due to the focus on the unchanged items. Also, it is probable that only a limited number of patients experience benefits in terms of active functional improvement following BTX-A injection, and that these numbers are diluted among patients who demonstrate no active functional improvement effect in large-scale clinical trials. Therefore, application of more focused outcome measures on the attainment of priority goals of functional improvement [13], or focused selection of target population is advocated. 

To overcome this limitation, the Goal Attainment Scale (GAS) has been recently adopted as a measurement tool to demonstrate the effect of BTX-A injection. This scale is more responsive and flexible across the domains of impairment, disability, and participation than the previously used standard instruments [11]. Goal setting has now become a routine part of practice in rehabilitation [20], and GAS has an advantage in terms of identifying individual unique goals by interviewing and negotiating with the patient for setting goals, for example, within an agreed date and defining expected goals that are practically feasible following intervention. However, there is considerable debate on the use of GAS in clinical practice [12] because GAS requires a physician to accurately identify the goal areas and the accurate scaling of the performance at follow-up after intervention [21]. Limitation in objectivity of GAS may also arise due to incongruency between the physician and the patient in the process of goal setting. Besides both goal setting and scale rating are reported to be very time-consuming for clinicians, making it less feasible for application in routine clinical practice [13,14], and many clinicians still feel more comfortable with the standardized measuring tools [13] 

Therefore, there is a need for an alternative method of measuring the effect of BTX-A injection on spasticity and functional improvement. To our knowledge, no report on the video clip analysis as an improved measurement tool following BTX-A injection for post-stoke upper limb spasticity has been published. This is the first feasibility study of video clip analysis as an outcome measure following BTX-A injection for post-stoke upper limb spasticity. To provide supporting evidence to demonstrate video clip analysis as an objective tool for evaluation of functional improvement following BTX-A injection, we tested the validity and interrater reliability of ratings by three blinded evaluators. The interrater agreement for video analysis was satisfactory, falling in the excellent range for both the hand motion (*k* = 0.857, ICC = 0.795) and gait associated hand posture (*k* = 0.924, ICC = 0.921). It was also feasible and time-cost effective in routine clinical practice, considering that video recording can be easily done during the routine physical and neurological examinations in the out-patient clinic without the need of a greater number of staff and more space, and, therefore, does not require extra time and cost for the patient and the clinician. Our results suggest that, although video clip analysis cannot be used as a primary outcome measurement tool, it is applicable as an alternative or additional secondary outcome measure for functional evaluation. We also suggest that video clip analysis is a useful and realistic evaluation tool following BTX-A injection that can be easily applied in private practice where resources such as time, evaluators, measuring instrument, and space are limited. 

There are a few limitations that warrant mention. First, we adopted a forced choice paradigm as to whether patients had improved or not, and regarded the post-injection video clips of more improved ones. Secondly, spontaneous recovery over time between the injection and follow-up should be taken into account as a partial explanation of the improvement, which could play an important role in improving function and reducing spasticity not necessarily due to the effect of BTX-A injection alone. In this study, the mean duration from injection to follow up was 4.21 weeks, which may not be sufficient to bring about the maximal change in either spasticity or functional improvement because timing of improvement of spasticity and active function may not coincide. Third, we did not compare video-clip analysis with standard spasticity grading results and did not grade the level of improvement from watching the video clip, such as much “improved” or “less improved”, so could not find compare pre- and post-treatment video images with pre- and post-questionnaires. However, we found that the patients who were mismatched for motion showed less change in MAS score than those who were matched, but without significance. 

Further evaluation on time-cost effectiveness of video clip analysis in comparison with the standard functional measurement tools or GAS would further contribute to validating the video clip analysis as a useful measurement tool. Demonstrating an effect of BTX-A injection for functional improvement was beyond the purpose of this study.

## 5. Conclusions

Many measurement tools are used for evaluation of the effects of BTX-A injection for post-stroke spastic upper limb function, but each has its limitations. Here, we suggest pre- and post-injection video clip analysis by physicians as a valid and reliable measure of improvement of function following BTX-A injection. Video clip analysis allows evaluation of the effects of BTX-A injection for post-stroke spastic upper limb function with high validity and reliability, and is useful, clinically feasible, efficient and less time consuming, even in a clinical setting with limited resources such as time, separate evaluators, measuring instruments, or space, as is often the case in private practices. 
